# Case report: A case of cutaneous anthrax guided by metagenomic next-generation sequencing technology

**DOI:** 10.3389/fmed.2024.1440130

**Published:** 2024-10-11

**Authors:** Lu Wang, Danli Wen, Yuanqing Qu, Qin Wang, Yuan Liu

**Affiliations:** Department of Laboratory Medicine, General Hospital of Western Theater Command of PLA, Chengdu, Sichuan, China

**Keywords:** *Bacillus anthracis*, cutaneous anthrax, metagenomic next-generation sequencing technology, infection, culture

## Abstract

Anthrax is an acute zoonotic infectious disease caused by *Bacillus anthracis*. It is categorized as a Class B (reported within 24 h of onset, including pulmonary anthrax, which is managed as a Class A infectious disease and reported within 2 h of onset) infectious disease in China. Human anthrax infection primarily occurs through direct or indirect contact with infected animals. This study reports a case of cutaneous anthrax where typical anthrax colonies were observed in conventional microbial cultures, and large Gram-positive rods with squared ends were visible under the microscope. The results from metagenomic next-generation sequencing (mNGS) suggested the presence of *Bacillus anthracis*. This research explores the value of combining traditional microbiology with mNGS technology for the early diagnosis and therapy of infectious diseases.

## Introduction

**Figure 1:**
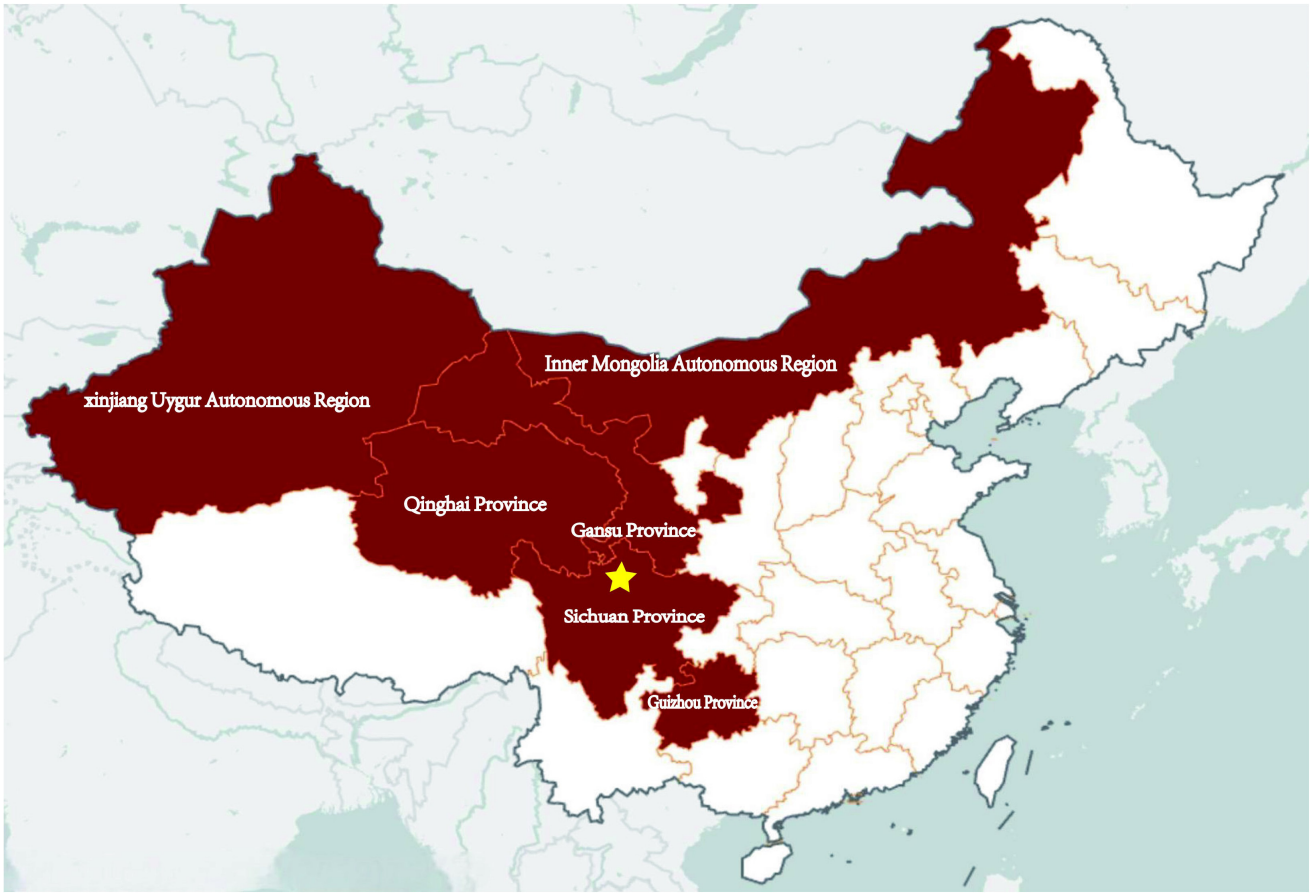
Map of China: the dark maroon areas indicate the provinces that are endemic for anthrax. The five-pointed star indicates where the patient lived and worked.

Anthrax is an ancient infectious disease, with the earliest records dating back to 80 AD (1). The threat posed by anthrax to both animal and human health is widely recognized, leading to the development and dissemination of extensive control measures for both animals and humans globally. Anthrax has nearly disappeared in developed countries (2). However, in developing countries, the disechanged. The clinical coursease still prevails in certain areas. It is estimated that there are between 20,000 to 100,000 (3). In China, cases of human anthrax are predominantly found in the western and northeastern regions, with 82% of the patients coming from the provinces or autonomous regions of Sichuan, Xinjiang, Gansu, Qinghai, Guizhou, and Inner Mongolia (4, 5) (Figure 1). From 2019 through 2023, there were 1,701 cases of anthrax reported in China, with a range of 200 to 500 cases per year (6).

Currently, few reports have described combining conventional microbiological methods with mNGS to diagnose and treat anthrax. The emergency department of our hospital recently admitted a patient from a pastoral area in Hongyuan County, the Aba Tibetan Autonomous Region, Sichuan Province, and this case is reported as follows.

## Case report

**Figure 2:**
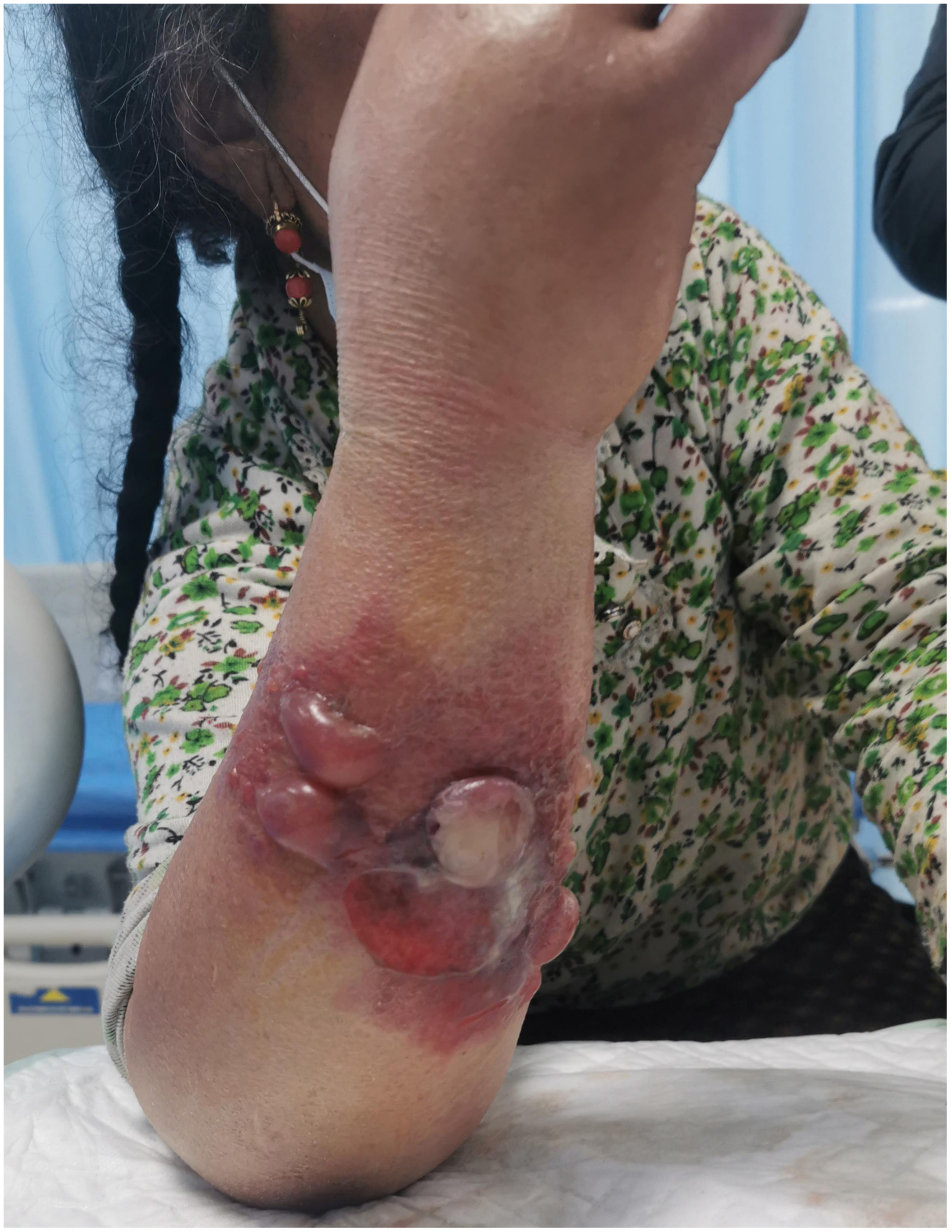
Patient's condition upon admission.

The patient, a 53-year-old Tibetan female from Hongyuan County, the Aba Tibetan Autonomous Region, Sichuan Province, was a herdsman with good past health, no bad habits, and no history of smoking or alcohol consumption. According to the patient, she developed small scratches on her arms while skinning a dead cattle on January 16, 2022. She noticed significant itching on her right forearm on January 28, 2022, and observed a red papule, which she did not regard it as serious. Subsequently, the rash gradually enlarged, forming vesicles containing pale yellow fluid and progressed to black eschars on January 29, 2022. There was notable swelling from the dorsum of the right upper arm to the axilla, accompanied by chills (Figure 2). She sought emergency care at our hospital on January 30.

On admission, T (Temperature) 36.9°C, P (Pulse) 89 beats/min, R (Breath) 16 breaths/min, BP (Blood Pressure) 122/75 mmHg. Height 155 cm, Weight 75 kg for a BMI (Body Mass Index) 31.21 kg/m^2^. Breath sounds of both lungs were clear; neither dry nor wet rales were heard. The abdomen was soft; there was no pain with pressure or rebound tenderness. Ultrasound of arterial blood vessels of the right upper limb showed no abnormalities. Laboratory tests were conducted on the patient (Table 1).

**Table 1:** The detailed results of the admission examination.

**Examination**		**Result**	**Reference values**
Routine blood tests	WBC	6.85 * 10^9^/L	3.50–9.50 * 10^9^/L
	RBC	6.77 * 10^12^/L	3.80–5.10 * 10^12^/L
	RBG	195g/L	115–150 g/L
	NE%	77.3%	40.0–75.0%
	LY%	16.1%	20.0–50.0%
Routine biochemical tests	HS-CRPQ	19.36 mg/L	0–3.0 mg/L
	GLU	7.53 mmol/L	3.80–6.10 mmol/L
	ALB	38.6 g/L	40.0–55.0 g/L
	ALT	35.8 IU/L	7.0–45.0 IU/L
	LDH	368.4 IU/L	95.0–245.0 IU/L

Upon presentation at our hospital, most vesicles on the patient's right upper arm had ruptured, leading to substantial exudation. The surrounding skin was discolored, and there was significant swelling from the dorsum of the right upper arm to the axilla. Despite the swelling, the patient complained of little pain and only slight itching. After considering her epidemiological history and ruling out other skin diseases with similar manifestations (e.g., furunculosis, cellulitis, and scrub typhus), a multidisciplinary team of consultants suggested there was a high probability the diagnosis was “cutaneous anthrax.” She was admitted to the Department of Infectious and Respiratory Diseases.

**Figure 3:**
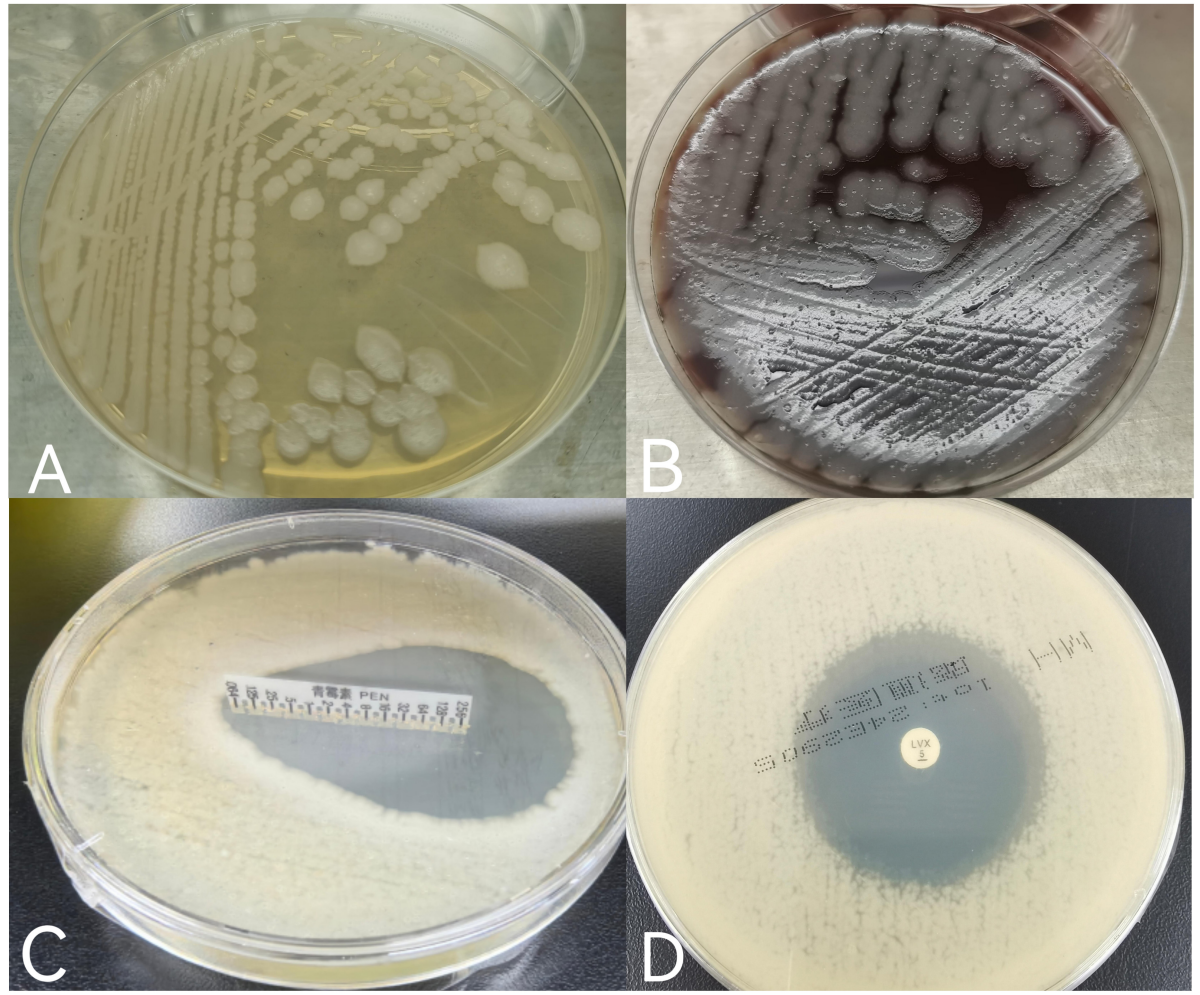
**(A)** Gray-white, opaque, circular or irregularly shaped colonies with a frosted glass appearance grown on ordinary nutrient agar plates; **(B)** gray-white, opaque, circular, or irregularly shaped colonies with a frosted glass appearance grown on Columbia blood agar plates; **(C)** the minimum inhibitory concentration (MIC) value of penicillin was < 0.064 μg/ml, and the drug sensitivity result was S (sensitivity) according to CLSIM45. **(D)** The inhibitory ring size of levofloxacin was 38 mm, and referred to the drug sensitivity folds of EUCAST2024, which was comprehensively judged as S (sensitivity).

The wound secretions were stained and cultured the day of arrival, January 30, 2022. Gram staining revealed large, thick Gram-positive rods with squared ends consistent with *Bacillus anthracis*. After 24 h of incubation in a normal incubator at 35°C, the culture produced gray-white, opaque, circular or irregularly shaped colonies with a frosted glass appearances (Figures 3A, B). By February 1, the isolate was determined to be sensitive to both penicillin (minimum inhibitory concentration [MIC] of 0.064 μg/ml) (7) and levofloxacin (inhibitory ring size of 38 mm) (8) (Figures 3C, D).

On January 31, pus aspirate from the patient's abscess was subjected to DNA extraction and to Metagenomic Next-Generation Sequencing (mNGS) to identify the causative infectious agent. The mNGS results returned on February 3 were as follows: phylum, Gram-positive bacteria, genus Bacillus, with 205 sequences, relative abundance 46.7%;species, Bacillus cereus group, with 146 sequences, relative abundance 33.26%, including 27 sequences for *Bacillus anthracis*.

**Figure 4:**
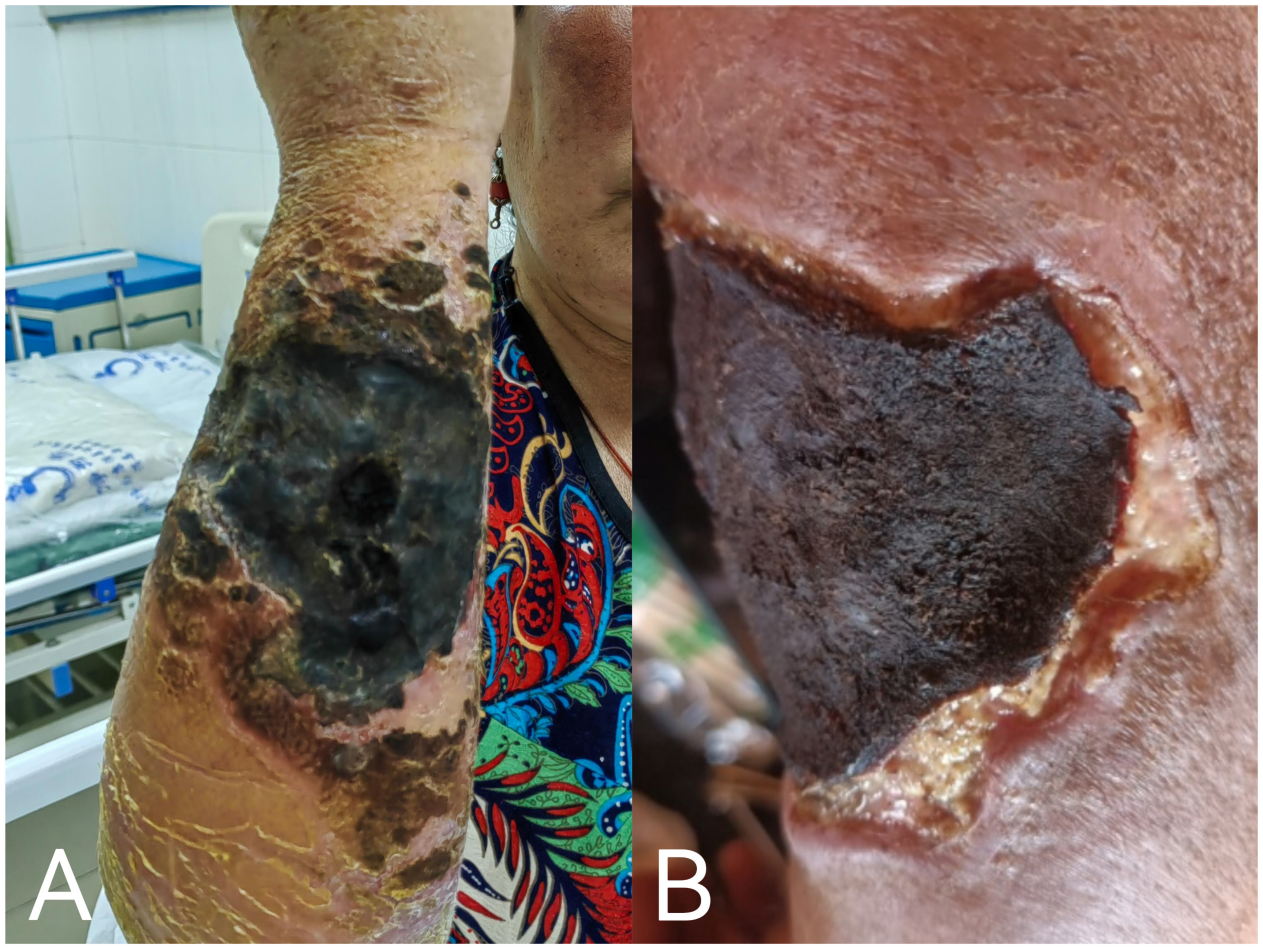
**(A)** By February 10, patient's lesion after treatment, with blisters gradually had merged and formed large eschar; **(B)** patient's condition at discharge on February 18.

A number of drugs may be used for cutaneous anthrax: the Ministry of Health of the People's Republic of China recommends penicillin G as the drug of choice (9), while the Sanford Guide recommends either doxycycline or ciprofloxacin (10). On clinicians' treatment experience and medication habits, in keeping with the local standard of care, the patient was treated with 800,000 IU of penicillin sodium intravenously every 8 h starting on January 30. On January 31, she received 40 mg of methylprednisolone sodium succinate intravenously every 12 h to reduce skin edema; 20 mg of esomeprazole magnesium enteric-coated capsules orally twice daily to prevent gastrointestinal ulcers; and potassium permanganate soaked for the skin, along with local dressing changed to suppress bacterial growth. After the above treatment, the patient's limb swelling was not significantly reduced, and there were still some new blisters on the right forearm and on February 3, the dosage was increased to 1, 600, 000 IU of penicillin sodium intravenously every 8 h. Additionally, 0.5 g of levofloxacin in sodium chloride was administered intravenously once daily to enhance anti-infective therapy. The patient's right upper limb showed a gradual decrease in swelling; by February 10, the blisters in the affected area had merged to form a large crust measuring approximately 15 cm by 10 cm (Figure 4A). After 18 days of anti-infective treatment, the swelling significantly reduced, and a black scab had formed at the site of the lesion, indicating effective treatment (Figure 4B). The patient was discharged on February 18 with instructions for home isolation and self-administered potassium permanganate soaked and dressing changed. The clinical course and medication use of the patient from admission to discharge for a total of 20 days is shown in (Supplementary Figure 1).

We did not find out the patient's follow-up information, contacted her by phone, according to the patient, no skin grafting was performed. The patient's lesion healed by about July, but the scar hyperplasia was large and deeply pigmented. She recovered full function of her arm, and resumed normal activities about 6 months after admission.

## Discussion

Anthrax is an acute zoonotic infectious disease caused by *Bacillus anthracis*. It is primarily divided into four types: cutaneous anthrax, inhalation anthrax, gastrointestinal anthrax, and injection anthrax (11). Cutaneous anthrax accounts for approximately 95% of all anthrax cases, and it usually occurs on exposed skin areas such as the face, neck, shoulders, hands, and feet, especially the hands (12). When anthrax bacilli or spores enter the body through broken skin, they proliferate locally, producing exotoxins, which cause swelling, hemorrhage, and tissue necrosis at the site of infection. The necrotic tissue and hemorrhagic exudate form a characteristic black crust, known as an eschar (13). Human infection typically occurs through contact with contaminated animal products, such as hides or meat. Herdsmen, veterinarians, slaughterhouse workers, animal caretakers, and those involved in processing animal hides and meats are at highest risk of infection due to their frequent contact with livestock (14). *Bacillus anthracis* can survive for extended periods in animal carcasses and in the environment, in particular in the soil, Anthrax spores are highly resistant, virulent, and proliferative, making the complete eradication of *Bacillus anthracis* spores exceedingly challenging (15). The incubation period of cutaneous anthrax is generally 2 to 3 days (9 to 12 h). It often takes only a few days for a patient to go from the appearance of a red rash to the formation of ulcers and black crusts (16). In this report, according to the patient's account, she had contacted with a dead cattle over 10 days prior to symptom onset, which likely died from an anthrax infection. It was an important cause of her infection. The period from exposure to symptom onset was over 10 days, indicating a longer incubation period.

Cutaneous anthrax is the most common form of the disease but remains extremely rare in clinical settings. Most medical professionals have limited opportunities to encounter cases, which easily leads to misdiagnosis and missed diagnosis. Differential diagnosis should be made from other infectious skin diseases such as furunculosis, erysipelas, streptococcal gangrene, cellulitis, cowpox, and orf (17). In order to prevent the disease from developing in a serious direction, an early and clear diagnosis is needed to make the correct treatment.

At present, etiological and serological tests are included in China's anthrax diagnosis and treatment protocol (2023 edition), including bacterial smears, bacterial cultures, nucleic acid testing, antigen testing, and antibody testing, while the mNGS technology is not mentioned. The result of bacterial culture could be affected by several factors, such as sample collection site, quality of microbial specimens, storage conditions, and transport time to the laboratory, potentially leading to false negatives and often necessitating repeated testing. Metagenomic next-generation sequencing (mNGS) is an emerging pathogenomic diagnostic technology. Metagenomic analysis involves high-throughput sequencing of nucleic acids extracted from biological specimens and bioinformatic comparison and analysis, providing information on the variety and abundance of microorganisms present. mNGS can be applied to a diverse range of samples, such as bronchoalveolar lavage fluid (BALF), tissue, sputum, pleural effusion, cerebrospinal fluid, pus, bone marrow, and nasal swabs (18–20), and it can detect almost all pathogens present in clinical samples (21), including bacteria, fungi, viruses, and parasites, within 48 h or even less (22). Most importantly, mNGS detected pathogens through DNA, allowing for successful detection regardless of whether the bacteria in the sample were alive or dead. It was valuable for patients to have been pre-treated with antibiotics, and have sterile cultures when they were hospitalized. In this case, the traditional microbiology results were consistent with the mNGS findings, which also confirmed the value of the mNGS technique in the diagnosis of anthrax.

Currently, penicillin G is the first-choice treatment for anthrax in China, only very few anthrax bacilli are resistant to penicillin G (9). Besides penicillin G, fluoroquinolone and tetracycline antibiotics are also effective against this bacterium (10). If cutaneous edema and rupture are likely to be complicated by *Staphylococcus aureus* infection, with methicillin-resistant *Staphylococcus aureus* (MRSA) detection rates as high as 30%, linezolid, which is effective against both MRSA and *Bacillus anthracis*, can be administered (23). For patients with malignant edema of the skin or severe cases, corticosteroids can be used to alleviate local edema and the development of septicemia (24). Additionally, topical applications of mirabilite combined with borneol can be used. Mirabilite—a traditional Chinese medicine whose main ingredient is sodium sulfate-containing water—reduces local leukocyte infiltration and thus the inflammatory response (25). Borneol—a traditional Chinese medicine which is the resin crystal of Lobeliaceae plant—has an analgesic effect and can inhibit bacterial infections such as *Staphylococcus aureus* and *Streptococcus* (26).

When treated with a variety of Abx (oral antibiotic) to which it is sensitive, cutaneous anthrax has a mortality rate below 2%. However, if left untreated, it has a high (25%) mortality rate. Any delay in treatment after onset of symptoms (either before or after hospitalization) increases the risk to the patient, It is necessary to improve the awareness of anthrax and make early differential diagnosis. The diagnosis of cutaneous anthrax often benefits from epidemiological evidence; however, due to the long survival of anthrax spores in nature, such evidence is difficult to obtain, and epidemiological evidence is not a requisite for diagnosis. Some patients may present with false-negative traditional microbiological results due to prior antibiotic use or inadequate sampling quality and prolonged transport time. Therefore, sequence analysis such as mNGS testing is suggested to be done on admission concurrently with attempts to culture and stain. Then if the cultures don't work because of something like pretreatment, there will be still an actionable result in a timely fashion. In this case, we increased a clinical treatment plan, raised the dose of penicillin and added levofloxacin in time. Rapid diagnosis and institution of appropriate antibiotics, combined with ongoing monitoring of the patient's condition can optimize the outcome of patients with this rare condition-cutaneous anthrax.

## Data Availability

The raw data supporting the conclusions of this article will be made available by the authors, without undue reservation.

## Supplementary material

Supplementary Figure 1 The clinical course and medication use of the patient from admission to discharge for a total of 20 days.

## References

[1] Li Z-P. Historical anthrax research and bacteriological warfare. Chin J Med Hist. (2002) 32:18. 10.3760/cma.j.issn.0255-7053.2002.01.02022227150

[2] Zheng C Ye J Song M Guo Y Gao W Hao J. The second cutaneous anthrax infection diagnosed by metagenomic next-generation sequencing: a case report. Medicine (Baltimore). (2024) 103:e36921. 10.1097/md.0000000000036921 38241573 PMC10798745

[3] Hugh-Jones M. 1996-97 global anthrax report. J Appl Microbiol. (1999) 87:189–91. 10.1046/j.1365-2672.1999.00867.x 10475945

[4] Chen WJ Li XL Fang LQ. Study on epidemiological features and spatial clusters of human anthrax in three provinces of western China from 2012 to 2013. J Pathogen Biol. (2016) 11:289–93. 10.13350/j.cjpb.160401

[5] Chen X Gao X-D Hu B-J Shi L Lin J-B. Prevention and control of nosocomial in fection of cutaneous anthrax under the guidance of metagenomic next-generation sequencing: a case report. Chinese J Clinical Medicine. (2020) 27:575–7. 10.12025/j.issn.1008-6358.2020.20201737

[6] Center for Disease Control and Prevention (CDC) [EB/OL]. Available at: https://www.phsciencedata.cn/Share/edtShareNew.jsp?id=39302 (accessed March 24, 2021).

[7] Clinical and Laboratory Standards Institute. Epidemiological Cut Off Values for Fastidious Bacteria Susceptibility Testing[S]. M45–ED3, CLSI (2016).32653660

[8] U.CAST. Breakpoint Tables for Interpretation of MICs and Zone Diameters (Version 7.1) The European Committee on Antimicrobial Susceptibility Testing (2024).

[9] He D. ≪Anthrax Diagnosis and Treatment protocol (2023 edition)≫ unscramble. Chinese J Antib. (2024) 49:749–54. 10.13461/j.cnki.cja.007716.9

[10] GilbertDN Saag MS Pavia AT Boucher HW. The Sanford guide to Antimicrobial Therapy 2023 (53rd Edition). Beijing: Peking Union Medical College Press (2024). p. 707

[11] Grunow R Verbeek L Jacob D Holzmann T Birkenfeld G Wiens D. Injection anthrax–a new outbreak in heroin users. Dtsch Arztebl Int. (2012) 109:843–8. 10.3238/arztebl.2012.0843 23267409 PMC3528063

[12] Swartz MN. Recognition and management of anthrax–an update. N Engl J Med. (2001) 345:1621–6. 10.1056/NEJMra012892 11704686

[13] Li B-P Shan B-H Zhu L Wu Y. Cutaneous anthrax: 3 cases report and literatures review. China J Leprosy Skin Dis. (2018) 34:146–9. 10.3760/cma.j.cmcr.2022.e0595238930622

[14] Sun Y Fu X-W Wu J-G Dong H-M Cun X-H Li X. Clinical imaging manifestations and diagnostic analysis of seven cases of anthrax. Electr J Emer Infect Dis. (2023) 8:49–52. 10.19871/j.cnki.xfcrbzz.2023.02.010

[15] Wang P Zhou W-Z Chen Q-R. Investigation on two anthrax outbreaks in Gannan prefecture of Gansu province. Bull Dis Control Prev. (2021) 36:21–3. 10.13215/j.cnki.jbyfkztb.200701530161194

[16] Zhang J Hou XY Wang JY Lu B. Case report: Cutaneous anthrax diagnosed using mNGS of a formalin-fixed paraffin-embedded tissue sample. Front Cell Infect Microbiol. (2024) 14:1329235. 10.3389/fcimb.2024.1329235 38638828 PMC11024221

[17] Lu R-J Liu S-L Wan X-F. Clinical analysis of 22 cases of cutaneous anthrax. Chin J Dermatovenereol. (2020) 34:899–902. 10.13735/j.cjdv.1001-7089.202001003

[18] Wang J Han Y Feng J. Metagenomic next-generation sequencing for mixed pulmonary infection diagnosis. BMC Pulm Med. (2019) 19:252. 10.1186/s12890-019-1022-4 31856779 PMC6921575

[19] Gu W Deng X Lee M Sucu YD Arevalo S Stryke D. Rapid pathogen detection by metagenomic next-generation sequencing of infected body fluids. Nat Med. (2021) 27:115–24. 10.1038/s41591-020-1105-z 33169017 PMC9020267

[20] Xiao YH Liu MF Wu H Xu DR Zhao R. Clinical efficacy and diagnostic value of metagenomic next-generation sequencing for pathogen detection in patients with suspected infectious diseases: a retrospective study from a large tertiary hospital. Infect Drug Resist. (2023) 16:1815–28. 10.2147/idr.S401707 37016633 PMC10066896

[21] Chiu CY Miller SA. Clinical metagenomics. Nat Rev Genet. (2019) 20:341–55. 10.1038/s41576-019-0113-7 30918369 PMC6858796

[22] Liu H Zhang Y Chen G Sun S Wang J Chen F. Diagnostic significance of metagenomic next-generation sequencing for community-acquired pneumonia in Southern China. Front Med (Lausanne). (2022) 9:807174. 10.3389/fmed.2022.807174 35242783 PMC8885724

[23] Dou M-Q Huangfu B-B Wang H-Y Lv X-D. Clinical pharmacists participated in pharmacy practice for a patientwith mixed Staphylococcus aureus infection and cutaneous anthraxwith malignant edematous China. Med Pharm. (2023) 13:174–8. 10.20116/j.issn2095-0616.2023.14.42

[24] Annane D Bellissant E Bollaert PE Briegel J Confalonieri M De Gaudio R. Corticosteroids in the treatment of severe sepsis and septic shock in adults: a systematic review. Jama. (2009) 301:2362–75. 10.1001/jama.2009.815 19509383

[25] Peng X-F Wang D-L Wang Y. Prevention and treatment of postoperative surgical site infection in abdominal surgery with external application of Chinese medicine mirabilite. Chin J Surg Integr Tradit Western Med. 2024:1–5. 10.3969/j.issn.1007-6948.2024.04.007

[26] Wu Y-F Zhu Z-Y Chen J-N Pi J-K Wei Wei Y-C Research progress on pharmacological effects of borneol and borneol ester. J Pharmac Res. (2020) 39:217–24. 10.13506/j.cnki.jpr.2020.04.007

