# Supplementary figures and images for: Case report: A case of cutaneous anthrax guided by metagenomic next-generation sequencing technology
Source: Front Med (Lausanne). 2024 Oct 11;11:1440130. doi: 10.3389/fmed.2024.1440130 (PMC11502367; doi:10.3389/fmed.2024.1440130)

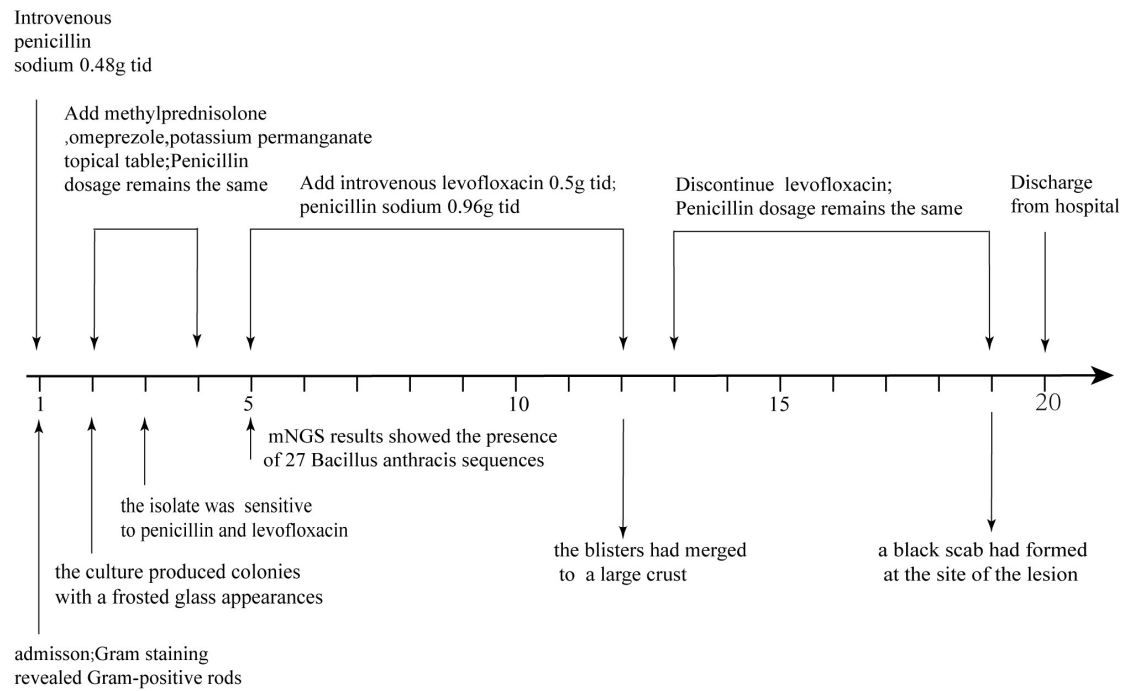

Hospital Days  
Figure 5

Supplement: Supplementary Figure 1 — The clinical course and medication use of the patient from admission to discharge for a total of 20 days. [file Image_1.pdf]
